# Successful conversion from butorphanol nasal spray to buprenorphine/naloxone using a low-dose regimen to assist with opioid tapering in the setting of chronic pain and migraine management in an older adult patient: A case report

**DOI:** 10.1080/24740527.2022.2090911

**Published:** 2022-08-18

**Authors:** Joshua MacAusland-Berg, Amy Wiebe, Radhika Marwah, Katelyn Halpape

**Affiliations:** a College of Pharmacy and Nutrition, University of Saskatchewan, Saskatoon, Saskatchewan, Canada; b USask Chronic Pain Clinic, University of Saskatchewan, Saskatoon, Saskatchewan, Canada; c Family Medicine, University of Saskatchewan, Saskatoon, Saskatchewan, Canada

**Keywords:** buprenorphine/naloxone, micro-dosing, chronic pain, butorphanol, older adult

## Abstract

**Background:**

Butorphanol is marketed as a treatment for migraines; however, evidence suggests that the harms of its use exceed the benefits. The short half-life of butorphanol places patients at high risk for opioid dependence and makes tapering a challenge. Buprenorphine/naloxone has unique pharmacological properties that are beneficial in chronic pain treatment. At this time there is limited published data on the use of micro-dosing initiation regimens in patients with chronic pain, especially in older adult patients.

**Aims:**

This article presents the case of an older adult patient for whom a buprenorphine/naloxone micro-dosing regimen was successfully utilized to aid discontinuation of butorphanol nasal spray, assist with opioid tapering, and manage chronic pain.

**Methods:**

This case took place in an outpatient setting while the patient was receiving care from an interprofessional chronic pain service. The electronic medical record was reviewed to obtain a summary of the case data. Informed patient consent was obtained.

**Results:**

We present a case of an older adult patient who had been using butorphanol nasal spray for migraine and general pain management for over 20 years. The risks of ongoing use of butorphanol (i.e., inter-dose-related pain, opioid dependence, possible opioid-induced hyperalgesia, and fall risk) no longer exceeded any perceived benefit. The patient was successfully transitioned onto sublingual buprenorphine/naloxone using a micro-dosing regimen.

**Conclusions:**

This case provides an example of the potential benefit buprenorphine/naloxone can have for patients with chronic pain and previous opioid exposure, especially older adults at risk of central adverse effects of opioids.

## RÉSUMÉ

**Contexte**: Le butorphanol est commercialisé comme traitement des migraines; cependant, les données probantes indiquent que les méfaits de son utilisation dépassent les avantages. La courte demi-vie du butorphanol expose les patients à un risque élevé de dépendance aux opioïdes et rend la réduction progressive un défi. La buprénorphine/naloxone possède des propriétés pharmacologiques uniques qui sont bénéfiques dans le traitement de la douleur chronique. À l’heure actuelle, il existe peu de données publiées sur l’utilisation de programmes d’initiation de microdosage chez les patients souffrant de douleur chronique, en particulier chez les patients adultes âgés.

**Buts**: Cet article présente le cas d’un patient adulte âgé pour lequel un programme de microdosage buprénorphine/naloxone a été utilisé avec succès pour aider à l’arrêt de la pulvérisation nasale de butorphanol, à la réduction progressive des opioïdes et à la prise en charge de la douleur chronique.

**Méthodes**: Ce cas s’est produit dans un cadre ambulatoire alors que le patient recevait des soins d’un service interprofessionnel de traitement de la douleur chronique. Le dossier médical électronique a été examiné pour obtenir un résumé des données du cas. Le consentement éclairé du patient a été obtenu.

**Résultats**: Nous présentons le cas d’un patient adulte âgé qui utilisait un vaporisateur nasal au butorphanol pour la migraine et la prise en charge générale de la douleur depuis plus de 20 ans. Les risques liés à l’utilisation continue du butorphanol (c.-à-d. la douleur interdose, la dépendance aux opioïdes, l’hyperalgésie possible induite par les opioïdes et le risque de chute) ne dépassaient plus les avantages perçus. Le patient a été transféré avec succès vers le buprénorphine/naloxone sublingual en utilisant un programme de microdosage.

**Conclusions**: Ce cas fournit un exemple de l’avantage potentiel de la buprénorphine/naloxone pour les patients présentant une douleur chronique et une exposition antérieure aux opioïdes, en particulier les personnes âgées à risque d’effets indésirables centraux des opioïdes.

## Introduction

Chronic pain is recognized as a disease by the World Health Organization and is defined as pain in one or more anatomical regions that lasts for longer than 3 months and is accompanied by significant emotional stress and/or functional disability.^1^ One in five Canadians live with chronic pain with the prevalence increasing in older adults.^1^ Patients with chronic pain are often prescribed different opioids, which can result in varying degrees of opioid tolerance, dependence, and adverse effects, including opioid-induced hyperalgesia and withdrawal mediated pain.^2–4^ Recent guidelines for chronic non-cancer pain indicate that long-term use of opioids for chronic pain should be limited given minimal benefit and high risk of adverse effects.^4^ Similarly, in the management of migraines, opioids are not recommended for long-term management.^5^

Butorphanol is a mixed agonist–antagonist opioid that was approved by the U.S. Food and Drug Administration in 1991 in a nasal spray formulation for short-term treatment of severe pain.^6^ However, it was later marketed for migraine treatment despite a paucity of evidence for this indication.^6^ Butorphanol was initially proposed to have less psychotomimetic effects and reduced abuse potential compared to other opioids; however, postmarketing surveillance data identified drastic increases in addiction-related adverse effects up to 24%.^6,7^ In 2013, the Canadian Headache Society strongly recommended against the use of butorphanol for migraines due to the risk of adverse effects, dependence, and medication overuse headaches; lack of evidence of benefit compared to other agents; and potential for withdrawal upon discontinuation.^7,8^ Unfortunately, this recommendation came after many patients had been started on butorphanol and possibly developed dependence, creating a need for strategies to safely transition patients off butorphanol.

Buprenorphine was developed for use as an analgesic in 1966; however, in recent years, buprenorphine combined with naloxone has been more commonly used in Canada for the management of opioid use disorder (OUD).^9^ Compared to full opioid agonists, the partial mu-receptor agonism of buprenorphine contributes to a lower risk of opioid-related adverse effects.^10^ It is postulated that the kappa receptor antagonism of buprenorphine is at least partially responsible for alleviating opioid-induced hyperalgesia.^11^ Buprenorphine is also unique in that its high-affinity binding and slow dissociation kinetics from the mu receptor contribute to milder withdrawal symptoms.^11,12^ Buprenorphine is thought to contribute to the reversal of opioid-induced hyperalgesia and reductions in opioid tolerance in the context of chronic pain.^10,12^ Several case studies have described successful use of buprenorphine/naloxone in patients with chronic pain and previous or long-term opioid exposure.^13–17^

In the setting of full opioid agonist use, initiation of buprenorphine requires careful consideration due to its high affinity for the mu-opioid receptor, which results in the displacement of other opioids from the receptor and can cause precipitated withdrawal.^10^ A traditional initiation regimen requires patients to be in moderate opioid withdrawal prior to buprenorphine/naloxone initiation to prevent sudden precipitated opioid withdrawal symptoms.^18^ Buprenorphine/naloxone low-dose or micro-dosing regimens have provided an additional strategy to initiate buprenorphine/naloxone without the patient needing to experience symptoms of withdrawal first.^19^ Several buprenorphine/naloxone micro-dosing case studies have been published, and each one used a slightly different regimen.^15–17,19^ These studies are largely surrounding the use of buprenorphine/naloxone in patients with OUD, and there remains a paucity of evidence on the use of micro-dosing buprenorphine/naloxone initiation regimens in patients with chronic pain, especially in older adults and in those using butorphanol for migraines.

We report a case of a buprenorphine/naloxone micro-dosing regimen successfully used to initiate therapy for an older adult patient with chronic, uncontrolled migraines and generalized body pain possibly related to opioid withdrawal.

## Materials and Methods

This is a case report of a patient who was referred to the USask Chronic Pain Clinic (CPC) by their family physician.^20^ The USask CPC’s electronic medical record for this patient case were retrospectively reviewed and summarized. Informed consent was obtained from the patient for publication of this case report.

## Results

### Background Patient Information

The patient was an 80-year-old female who lived with her husband in their own home. She was independent for all activities of daily living; however, her pain did interfere with her ability to optimally complete some household chores. Over several years, she had become increasingly concerned with her regular use of, and dependence on, butorphanol nasal spray and was referred to the USask CPC by her family physician for assistance in reducing and stopping this medication.

Her past medical history included migraines that started at the age of 24; chronic pain due to a hernia repair, lower back pain triggered by activity, and a potential fibromyalgia diagnosis; dyslipidemia; a previous gastric ulcer; and a renal cyst. One month prior to her initial assessment she developed a new pain between her shoulder blades. Renal and hepatic function were normal for her age. Substance use history was positive for smoking ten or fewer tobacco cigarettes per day. A urine toxicology screen was not performed. Her medication regimen included butorphanol 10 mg/mL nasal spray (sometimes diluted with water) as needed, candesartan/hydrochlorothiazide 16 mg/12.5 mg by mouth (PO) daily, rosuvastatin 20 mg PO every evening, simethicone 180 mg PO twice daily as needed (BID PRN), zinc 50 mg PO daily, vitamin C 1000 mg PO daily, and vitamin D 2000 units PO daily.

At the time of initial assessment, she had been using the butorphanol nasal spray for approximately 20 years for treatment of migraines and progressively also for her other chronic pain conditions. The patient had high levels of anxiety and guilt regarding her physical and emotional dependence on butorphanol, which were exacerbated by the challenge to find a health care provider to support discontinuation from butorphanol. At the initial assessment she was using ten bottles (250 mg) of butorphanol over a 3- to 4-week period, which cost approximately CA$600 per month. Prior to this she had been using twice the amount of butorphanol (500 mg over 3 to 4 weeks). In an additional attempt to reduce her butorphanol use, she sometimes diluted her butorphanol approximately half and half with water, which resulted in uncertainty and lack of consistency in the dose being administered. The exact details regarding previous dose reductions at the initial assessment were unknown because all were done as a self-directed strategy prior to her involvement with the USask CPC. The patient stated that she was pain-free after taking a dose of butorphanol, including resolution of the new pain between her shoulder blades, but had experienced instances where she ran out of butorphanol, which resulted in withdrawal symptoms such as stomach pain, headache, nausea, dry heaving, and a sensation of restless legs.

### Patient Assessment

Several measurement-based care tools that were completed as part of the patient’s initial assessment with the USask CPC are summarized in Table 1. The patient’s Prescription Opioid Misuse Index was positive suggesting, a need for further assessment of a possible OUD diagnosis.^21^ Her Central Sensitization Inventory was suggestive of the presence of mild central sensitization (i.e., nociplastic pain).^22^ The Brief Pain Inventory scores indicated that the average intensity was low; however, pain severity was 8/10 at its worst and 6/10 on average, which impacted some aspects of the patient’s daily activities.^23^ The patient’s Douleur Neuropathique en 4 questionnaire score, which assesses for neuropathic pain, was 3/10. This score suggests that neuropathic pain was not a major problem for this patient.^24^ The patient’s Pain Catastrophizing Scale score, which assesses a patient’s thoughts about their pain experience, was 15/52. A positive score on the Pain Catastrophizing Scale is above 30; thus, this patient did not score positively.^25^

**Table 1.** Initial assessment scores.

Scoring tool	Score
Brief Pain Inventory–Severity	3.5/10
Central Sensitization Inventory	33/100
Douleur Neuropathique 4 (clinical exam not performed)	3/10
Pain Catastrophizing Scale	15/52
Prescription Opioid Misuse Index	3/6

It was determined that butorphanol was contributing more harm (i.e., possible opioid-induced hyperalgesia, inter-dose withdrawal pain, physical tolerance, possible OUD) than benefit. The patient was thought to be experiencing inter-dose withdrawal pain based on the new onset of pain between her shoulders that started when she self-reduced her butorphanol dose and was alleviated when she took a butorphanol dose. This inter-dose withdrawal pain combined with the short half-life of butorphanol made further tapering butorphanol a suboptimal option. Additionally, the patient was highly motivated to make a change to her pharmacotherapy for pain. Thus, the decision was made to transition to buprenorphine/naloxone using a micro-dosing initiation regimen to minimize the risk of opioid withdrawal symptoms and pain exacerbation.

### Buprenorphine/Naloxone Initiation

Buprenorphine/naloxone was initiated at 0.5 mg/0.125 mg sublingual (SL; one quartered 2 mg/0.5 mg tablet) daily and escalated as presented in Table 2. The target dose of buprenorphine/naloxone was set to be 2 mg/0.5 mg SL BID, at which point the patient was to discontinue the butorphanol nasal spray. The buprenorphine/naloxone was provided in compliance packaging to allow for an at home micro-dosing initiation. Clonidine was also initiated at 0.05 to 0.1 mg PO BID as needed to be used for opioid withdrawal for 2 weeks during the transition from butorphanol to buprenorphine/naloxone. The patient expressed concerned with management of acute migraines, should they occur, and was also prescribed sumatriptan 20 mg nasal spray to be used in one nostril as needed. The patient was advised to only use this medication in the case of an acute migraine. She was also instructed to monitor the number of doses taken per month and to limit doses to a recommended maximum of nine doses per month.

**Table 2.** Buprenorphine/naloxone regimen (Using micro-dosing for initiation).

Day(s)	Buprenorphine/naloxone SL dose (buprenorphine 2 mg/naloxone 0.5 mg tabs)	Butorphanol 10 mg/mL nasal spray
1	0.5 mg/0.125 mg once daily	Maintained dose at time of initial assessment (approximately five 1 mg sprays per day)
2	0.5 mg/0.125 mg twice daily	
3	1 mg/0.25 mg twice daily	
4	2 mg/0.5 mg twice daily	
5	2 mg/0.5 mg twice daily	Discontinued
6–25	2 mg/0.5 mg twice daily	

During the buprenorphine/naloxone initiation, close communication was maintained between the USask CPC pharmacist and the patient’s community pharmacy. The community pharmacist was initially hesitant to dispense the butorphanol when the patient had run out during the buprenorphine/naloxone initiation. This reluctance was potentially due to a misunderstanding of the timeline and purpose of the micro-dosing initiation. With additional communication between the USask CPC pharmacist and the community pharmacists, these issues were rectified with no delay, and the patient continued with the buprenorphine/naloxone micro-dosing initiation regimen as planned.

Transition to the buprenorphine/naloxone was generally well tolerated following the initiation regimen in Table 2; however, a mild pruritic rash appeared on the patient’s feet, hands, and scalp. The patient also experienced some mild withdrawal symptoms immediately following the discontinuation of butorphanol on day 5, which she described as abnormal nighttime dreams, followed by nausea and dry heaving upon awakening. She took a single dose of clonidine 0.1 mg PO, which resulted in complete cessation of these withdrawal symptoms. There was no increase in pain, and she did not require the use of the sumatriptan nasal spray for migraine during the initiation. Once at the target buprenorphine/naloxone dose of 2 mg/0.5 mg SL BID, although the patient’s pain was adequately managed, she did experience some daytime sedation.

### Buprenorphine/Naloxone Dose Reduction

Due to the presence of daytime sedation and itchiness, the patient expressed a strong preference to reduce the dose of buprenorphine/naloxone with the goal of eventual discontinuation. The tapering timeline is shown in Table 3. To accommodate the patient’s request for a relatively rapid taper, dose reductions were made approximately every 2 weeks. While dose reductions were in progress, the patient was instructed to self-monitor for changes such as the development of opioid withdrawal symptoms, worsening pain, daytime sedation, status of pruritic rash, and number of sumatriptan doses she required. When buprenorphine/naloxone was reduced to 0.5 mg/0.125 mg SL once daily the patient experienced significant worsening of migraines and onset of withdrawal symptoms. Therefore, the dose was elevated back to buprenorphine/naloxone 1 mg/0.25 mg SL once daily, which adequately managed the patient’s pain, migraines, and withdrawal symptoms. The patient was satisfied with this regimen and was subsequently discharged from USask CPC at this dose, with the possibility of future further tapering potentially to discontinuation of the buprenorphine/naloxone by her family physician after a period of stability at the 1 mg/0.25 mg dose.

**Table 3.** Summary of buprenorphine/naloxone tapering and corresponding clinical status.

Buprenorphine/naloxone sublingual dose	Days at dose	Clinical status	Number of sumatriptan doses
2 mg/0.5 mg twice daily	19	Rash/itch on patient’s back, feet, and scalp. Managed with two doses of diphenhydramine 25 mg. Rash was noticed after 7 days at target dose.	0
2 mg/0.5 mg AM + 1 mg/0.25 mg PM daily	14	Rash improved 15 days after dose reduction. No withdrawal symptoms identified.	1
1 mg/0.25 mg twice daily	14	Rash/itch almost completely gone. Not a concern for the patient. No withdrawal symptoms identified.	2
1 mg/0.25 mg daily	20	No withdrawal symptoms identified. Moderate constipation managed with polyethylene glycol 3350 daily. Started acetaminophen 500 mg PRN for mild headache.	2
0.5 mg/0.125 mg daily	7	Dramatic increase in migraine frequency. Potential withdrawal symptoms identified (i.e., generally feeling unwell).	6
1 mg/0.25 mg daily	15	Mild headache that did not impact patient’s ability to function. No withdrawal symptoms identified. Clonidine prescription returned to pharmacy for disposal.	1

Overall, the patient was successfully converted from butorphanol nasal spray to buprenorphine/naloxone 2 mg/0.5 mg SL BID using a micro-dosing initiation regimen with minimal opioid withdrawal symptoms and no pain exacerbations. At the patient’s request, and to find the lowest effective dose, buprenorphine/naloxone was reduced at 12- to 15-day intervals to a dose of 0.5 mg/0.125 mg SL at bedtime which resulted in an increase in migraine frequency and severity. The dose was then returned to 1 mg/0.25 mg SL QHS, because this dose adequately managed her pain and migraines, without causing daytime sedation or rash. The patient’s medications at discharge included buprenorphine/naloxone 1 mg/0.25 mg SL every evening, sumatriptan 20 mg nasal spray daily as needed, acetaminophen 500–1000 mg PO daily as needed, polyethylene glycol 3350 17 g PO daily, rosuvastatin 20 mg PO every evening, and vitamin D 2000 units PO daily. The initiation of buprenorphine/naloxone provided substantial improvements to the patient’s quality of life. This was identified as the near elimination of her generalized and chronic pain, as well as reduced migraine frequency, which allowed her to complete daily activities without interruption. The patient was extremely satisfied with this change.

### Discussion

We present a case of an 80-year-old female who was successfully transitioned to buprenorphine/naloxone from butorphanol using a micro-dosing initiation regimen.

It is possible that this patient’s prolonged history of butorphanol nasal spray use was contributing to opioid-induced hyperalgesia as well as inter-dose withdrawal pain and led to the development of an OUD. Despite its unique mechanism of action, animal studies indicate that butorphanol can result in opioid-induced hyperalgesia.^26^ The patient stated that the butorphanol spray did relieve her pain immediately after use; however, she often experienced increased pain throughout the day between doses. Butorphanol possesses a half-life of 5 to 6 h; thus, the patient’s worsening pain could have been a symptom of opioid withdrawal.^27^ Both the prolonged half-life of buprenorphine and its proposed ability to alleviate hyperalgesia in patients with previous opioid exposure could have contributed to the benefit seen in this patient.^10−12^ Furthermore, the elimination of chronic back pain following conversion to buprenorphine/naloxone provided the patient increased clarity to better identify potential migraines and differentiate them from mild headaches, which allowed her to initiate proper migraine pharmacological management.

Butorphanol has variable estimations in its equivalence to morphine that range from 10 to 15 times more potent than morphine for oral formulations.^27^ This, combined with how the patient was diluting her butorphanol with water, made it impossible to definitively estimate her daily morphine equivalent dose, especially because she did not consistently dilute the butorphanol each day. However, it was estimated that her daily consumption of butorphanol was approximately less than 100 morphine equivalents at time of her initial assessment. This was based on a usually daily dose of five butorphanol sprays per day. A single spray would contain 1 mg butorphanol (undiluted) or 0.5 mg butorphanol (diluted); thus, she was using 2.5–5 mg intranasal butorphanol per day. The butorphanol product monograph indicates that 2 mg intravenous butorphanol is equal to 10 mg of intravenous morphine.^28^ The bioavailability of intranasal butorphanol in elderly patients is 48%; thus, approximately 1 mg of intranasal butorphanol is equal to 10 mg of intravenous morphine or 30 mg of oral morphine.^28,29^ However, given the unique agonist–antagonist effects of butorphanol, it is challenging to make direct conversions to morphine. Similarly, it is challenging to convert between buprenorphine and morphine, given the unique partial agonist properties of buprenorphine. Buprenorphine to morphine equianalgesic ratios range between 1 to 25 and 1 to 110; thus, a mid-range ratio of 1 to 75 would equate to 0.4 mg SL buprenorphine is equal to 30 mg oral morphine.^30–32^ Due to the lack of fixed morphine equivalent dose for buprenorphine, when converting to buprenorphine from a full opioid agonist a common approach is to titrate the buprenorphine dose based on clinical response.^13–17^

Given the estimated morphine milligram equivalent dose the patient was using, the challenges in calculating equivalent doses with butorphanol and buprenorphine, and the patient’s chronic pain conditions and advanced age, the initial target dose for buprenorphine/naloxone was set lower than other buprenorphine/naloxone micro-dosing initiation regimens.^15–17^ Additionally, a suggested micro-dosing regimen for conversion to buprenorphine from short-acting opioids was used to guide the transition.^33^

In the presented case, butorphanol was discontinued on day 5 of the micro-dosing regimen (Table 2), which differs from some micro-dosing regimens in which the preexisting opioid is discontinued on day 7.^12,14^ However, there are micro-dosing regimens that recommend discontinuing opioids on day 5 when the opioid is an immediate release formulation, which was the rationale for this case.^33,34^

Following stabilization at buprenorphine/naloxone 2 mg/0.5 mg SL BID the patient developed a pruritic rash. The rash, combined with the patient’s high level of motivation to reduce overall daily opioid requirements, prompted attempts to identify the lowest effective dose. It is important to note that dose reductions were performed at a more accelerated rate than the USask CPC pharmacist would have typically recommended due to how motivated the patient was to reduce opioid requirements, especially given the presence of daytime sedation and rash. The rash had almost completely dissipated at a dose of buprenorphine/naloxone 1 mg/0.25 mg SL BID, which indicates that the rash could have been caused by higher doses of the buprenorphine/naloxone; however, this is not a certainty, because the rash could have dissipated on its own if the higher dose was maintained for a longer period. Additionally, rash is a relatively rare side effect caused by buprenorphine/naloxone appearing in approximately 5% of patients.^18^

In Canada, buprenorphine/naloxone is currently only indicated for use in patients with OUD, which resulted in some confusion in the management of this case from community pharmacy perspective. This was resolved through detailed communication regarding the indication for buprenorphine/naloxone as well as the purpose and timeline of the micro-dosing initiation regimen. This exemplifies the importance of proper communication among health care professionals given the relatively limited evidence for off-label use of buprenorphine/naloxone for chronic pain, especially when initiated using a unique micro-dosing regimen.

This case provides an example of the potential benefit buprenorphine/naloxone can have for patients with chronic pain and long-term opioid exposure, especially elderly adults at risk of central adverse effects of opioids. Although long-term opioids are not recommended for chronic non-cancer pain or migraine management, buprenorphine/naloxone does provide a therapeutic strategy to assist with opioid tapering and to reduce overall opioid-related risk in situations when complete cessation of opioids may not be feasible due to the existence of opioid dependence or an OUD. Additionally, this case highlights a unique micro-dosing initiation regimen in which the patient’s baseline opioid medication, butorphanol, was successfully discontinued after only 4 days of the micro-dosing initiation regimen.
